# Calcium intake, calcium supplementation and cardiovascular disease and mortality in the British population: EPIC-norfolk prospective cohort study and meta-analysis

**DOI:** 10.1007/s10654-020-00710-8

**Published:** 2020-12-31

**Authors:** Tiberiu A. Pana, Mohsen Dehghani, Hamid Reza Baradaran, Samuel R. Neal, Adrian D. Wood, Chun Shing Kwok, Yoon K. Loke, Robert N. Luben, Mamas A. Mamas, Kay-Tee Khaw, Phyo Kyaw Myint

**Affiliations:** 1 Ageing Clinical and Experimental Research (ACER) Team, University of Aberdeen, Aberdeen, UK; 2 Aberdeen Diabetes and Cardiovascular Centre, School of Medicine, Medical Sciences and Nutrition, University of Aberdeen, Room 4:013, Polwarth Building, Foresterhill, Aberdeen, Scotland AB25 2ZD UK; 3 Department of Epidemiology, School of Public Health, Iran University of Medical Sciences, Tehran, Iran; 4 Endocrinology Research Centre, Institute of Endocrinology and Metabolism, Iran University of Medical Sciences, Tehran, Iran; 5 Keele Cardiovascular Research Group, Keele University, Stoke-on-Trent, UK; 6 Norwich Medical School, University of East Anglia, Norwich, UK; 7 Department of Public Health and Primary Care, University of Cambridge, Cambridge, UK

**Keywords:** Dietary calcium, Calcium supplements, Cardiovascular disease, Systematic review, Meta-analysis, Mortality

## Abstract

The role of dietary calcium in cardiovascular disease prevention is unclear. We aimed to determine the association between calcium intake and incident cardiovascular disease and mortality. Data were extracted from the European Prospective Investigation of Cancer, Norfolk (EPIC-Norfolk). Multivariable Cox regressions analysed associations between calcium intake (dietary and supplemental) and cardiovascular disease (myocardial infarction, stroke, heart failure, aortic stenosis, peripheral vascular disease) and mortality (cardiovascular and all-cause). The results of this study were pooled with those from published prospective cohort studies in a meta-analsyis, stratifying by average calcium intake using a 700 mg/day threshold. A total of 17,968 participants aged 40–79 years were followed up for a median of 20.36 years (20.32–20.38). Compared to the first quintile of calcium intake (< 770 mg/day), intakes between 771 and 926 mg/day (second quintile) and 1074–1254 mg/day (fourth quintile) were associated with reduced all-cause mortality (HR 0.91 (0.83–0.99) and 0.85 (0.77–0.93), respectively) and cardiovascular mortality [HR 0.95 (0.87–1.04) and 0.93 (0.83-1.04)]. Compared to the first quintile of calcium intake, second, third, fourth, but not fifth quintiles were associated with fewer incident strokes: respective HR 0.84 (0.72–0.97), 0.83 (0.71–0.97), 0.78 (0.66–0.92) and 0.95 (0.78–1.15). The meta-analysis results suggest that high levels of calcium intake were associated with decreased all-cause mortality, but not cardiovascular mortality, regardless of average calcium intake. Calcium supplementation was associated with cardiovascular and all-cause mortality amongst women, but not men. Moderate dietary calcium intake may protect against cardiovascular and all-cause mortality and incident stroke. Calcium supplementation may reduce mortality in women.

**Electronic supplementary material:**

The online version of this article (10.1007/s10654-020-00710-8) contains supplementary material, which is available to authorized users.

## Introduction

The role of dietary calcium intake and calcium supplementation in cardiovascular disease (CVD) prevention remains controversial [1]. Whilst some studies advocate that higher calcium intake may be associated with higher mortality [2, 3], others suggest the contrary [4, 5]. Furthermore, conflicting evidence exists regarding calcium supplementation and its effects on important determinants of adverse cardiovascular outcomes. Whilst there is evidence to suggest that increased calcium intake is associated with improved lipid profiles [6] and reduced blood pressure [7], increased serum calcium levels have also been associated with an increase in arterial calcification [8]. Furthermore, several Mendelian randomisation studies have underlined the relationship between increases in serum calcium levels and increased incidence of coronary artery disease [9] and myocardial infarction [9, 10], but not ischaemic stroke [11]. Despite several previous systematic reviews and meta-analyses having analysed this relationship, these have yielded conflicting results in terms of both incident cardiovascular disease (CVD) and mortality [12–16].

To our knowledge, no prospective cohort studies have examined the relationship between calcium intake or supplementation and incident cardiovascular disease or mortality in the British population over very long-term follow-up.

In view of this literature gap, we utilised data from European Prospective Investigation into Cancer (EPIC)-Norfolk prospective population-based study to evaluate the association between calcium intake (dietary intake and supplement use) and mortality and incident CVD over very long follow-up in excess of 20 years and incorporate these results into the current body of evidence by performing an updated meta-analysis.

## Methods

### Participants

Participants were selected from the European Prospective Investigation into Cancer, Norfolk (EPIC-Norfolk) prospective cohort study. Study recruitment methods have been described in detail previously [17]. In brief, men and women aged 40–79 (at study baseline) from 35 participating General Practices in Norfolk, England were invited to participate. This study was approved by the Norwich Local Research Ethics Committee. All participants provided signed informed consent.

### Dietary data collection

Dietary calcium and vitamin D intake were estimated from a food frequency questionnaire (FFQ) which contained a list of 130 foods and was designed to measure the usual food intake of participants during the previous year. Additional questions gathered specific information on the type and amount of cereal, fat and milk consumed and the use of any vitamin or mineral supplements (including calcium supplements).

### Non-dietary data collection

Non-dietary data were collected from a health and lifestyle questionnaire (HLQ) and by the means of a health check. Smoking history was obtained using the questions “Have you ever smoked as much as one cigarette a day for as long as a year?” and “Do you smoke cigarettes now?”; education level was recorded as no qualification, O-level, A-level or degree or higher qualification; and social class was classified according to the Registrar General’s occupation-based classification scheme [18, 19]. Medical history was determined using the question “Has a doctor ever told you that you have any of the following?” followed by a list of medical conditions including high blood pressure, cancer, heart attack, stroke and diabetes. In addition, a four-level physical activity index was derived from the validated EPIC short physical activity questionnaire designed to assess combined work and leisure activity [20].

At the health check, trained nurses measured height, weight, blood pressure and collected blood samples. Weight was measured to the nearest 0.2 kg using digital scales (Salter, UK) with participants wearing light clothing without shoes, and height was measured to the nearest 0.1 cm using a stadiometer with shoes removed. Body mass index (BMI, kg/m^2^) was calculated as weight (kg) divided by the square of the height (m^2^). Blood pressure (BP) was measured with an Accutorr sphygmomanometer (Datascope, UK) after the participant had been seated for 3 min, and the mean of two BP measurements was used for analysis. Serum total cholesterol was measured from non-fasting venous blood samples with an RA 1000 (Bayer Diagnostics, Basingstoke, UK). A total of 25,639 participants completed all baseline components and attended the health check.

### Outcomes

International Classification of Disease 10 (ICD-10) codes were used to define the outcomes for the study: all-cause mortality, cardiovascular mortality, incident all cardiovascular disease, incident aortic stenosis, incident heart failure, incident myocardial infarction, incident peripheral vascular disease and incident stroke. Cardiovascular events evaluated were myocardial infarction (I21–22), stroke (I60–I69), heart failure (I50), aortic stenosis (I35), peripheral vascular disease (I70–79). The composite cardiovascular disease outcome was defined as I10–79. In addition, data from the Office of National Statistics were used to ascertain all-cause mortality (up to March 2016). Cardiovascular mortality was defined as death with a cause of death listed as I10–79. Furthermore, all participants were linked to National Health Service hospital information system and ENCORE (East Norfolk Commission Record) to allow notification of any hospital admission. While validation studies have not been performed for all the diagnoses and outcomes from EPIC-Norfolk included in this study, previously published validation studies of random samples from EPIC-Norfolk assessing the diagnoses of stroke [21], heart failure [22] and the physical activity questionnaire [20] have shown that these parameters were ascertained with high accuracy. These studies overall suggest the high validity of the diagnoses ascertained in the EPIC-Norfolk cohort. Furthermore, the United Kingdom National Health Service (NHS) captures almost all events as everyone in the UK is registered with a General Practitioner and has an associated NHS number. Therefore, data capture using NHS record linkage system is extremely robust.

### Statistical analysis

Data were analysed using Stata 15.1 SE (StataCorp 2017, Stata Statistical Software: Release 15, College Station, TX: StataCorp LLC). Participants with missing or invalid covariate data and those with prevalent cancer or cardiovascular disease (stroke, myocardial infarction) were excluded. Participants were categorised by quintiles of dietary calcium intake in order to allow comparison with the results yielded by previous nutritional epidemiology studies as well as to allow modelling of non-linear relationships between calcium intake and outcomes whilst providing clinically meaningful thresholds of recommended intake. Participants were also dichotomised by usage of calcium supplements (use vs. no use). Baseline characteristics were compared between groups using the *χ*^2^ test and one-way ANOVA for categorical and continuous variables, respectively.

#### Primary analyses

Cox proportional hazard models were used to calculate the risk of mortality (all-cause and cardiovascular) associated with calcium exposure. Models were constructed quantifying the association between: (1) calcium exposure (as quintiles of calcium intake) and pre-specified outcomes and (2) use of calcium supplements and pre-specified outcomes. Multivariable adjustments were chosen based on clinical judgement and previous literature [2–5, 23–25]. All analyses were adjusted for demographic data (age, sex), biological factors (BMI, systolic blood pressure, low-density lipoprotein, high-density lipoprotein and total cholesterol levels), social class, education level, lifestyle factors (physical activity, alcohol intake, smoking status—current/former/never smoker), pre-existing comorbidities (hypertension, diabetes), medication and supplement use (aspirin, statins, ACE inhibitors, beta-blockers, angiotensin receptor blockers, vitamin supplement use, calcium supplement use),dietary intake (total energy intake, fruit and vegetable, vitamin D) and current/former usage of hormone replacement therapy (HRT).

#### Secondary analyses

Secondary analyses were also performed in order to evaluate the sex-stratified relationship between quintiles of calcium intake and the pre-specified outcomes. Furthermore, the relationship between quintiles of calcium intake and the pre-specified outcomes was also evaluated among (1) participants without prevalent hypertension or diabetes mellitus at baseline and (2) female participants ever having used HRT at baseline. Finally, in order to evaluate the non-linear associations between calcium intake and the pre-specified outcomes, restricted cubic splines (RCSs) were used to model the hazard ratio associated with calcium intake as a continuous variable for each outcome as a flexible non-linear function, where appropriate. The Akaike Information Criterion was calculated for the linear model and for RCS models with varying degrees of freedom (df = 2 to df = 7). The model with the lowest AIC was chosen for each outcome. Where an RCS model had a lower AIC than the linear model, the likelihood-ratio test was used to confirm that this RCS model provides a better fit for the data than the linear model. Supplementary Table 1 details the best fitting models for each regression. The mean calcium intake (1018.83 mg/day) was used as reference for all analyses.

### Meta-analysis

A systematic search on Medline was conducted up to September 2020 for prospective cohort studies that evaluated the association between calcium dietary intake or supplementation and cardiovascular mortality or all cause-mortality. The following search terms were utilised: calcium [ti] AND ((“cardiovascular system”[MeSH Terms] OR (“cardiovascular”[All Fields] AND “system”[All Fields]) OR “cardiovascular system”[All Fields] OR “cardiovascular”[All Fields]) OR (“stroke”[MeSH Terms] OR “stroke”[All Fields])) AND (diet * OR supplementv* OR intake). In addition to the studies thus identified, we also included all studies that had been included in previously published systematic reviews and meta-analyses [12–15].

**Fig. 1:**
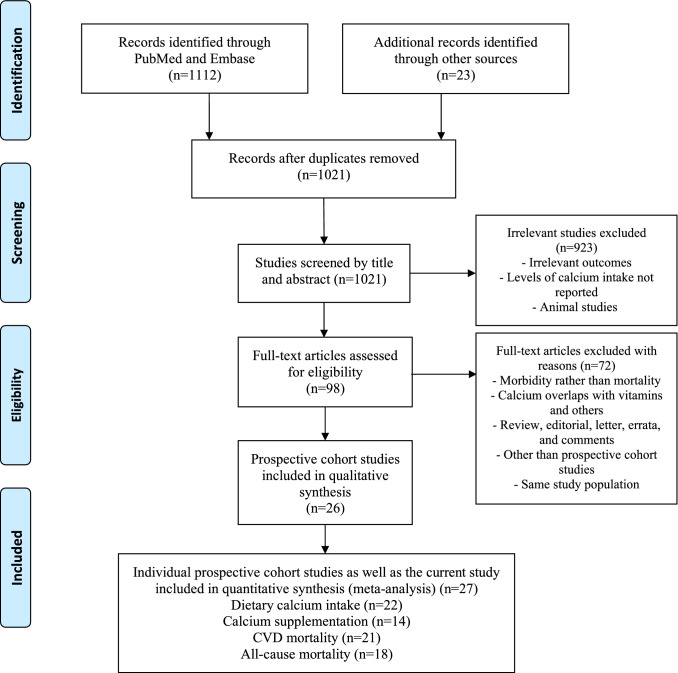
PRISMA Flow Diagram

We conducted an updated systematic review and meta-analysis evaluating the association between high dietary calcium intake or calcium supplementation and mortality (cardiovascular and all-cause). We included in the meta-analyses all eligible studies identified by the literature search, those identified by the reference analysis of previously published meta-analyses, as well as the current study. Figure 1 details the PRISMA flow diagram. We have thus identified 26 prospective cohort studies with a total of 1,828,149 participants [2–5, 23–44]. Details of the studies characteristics and results are shown in Supplementary Tables 2 and 3.

The meta-analysis was performed using STATA version 13.0 (College Station, TX). Pooled relative risk (RR) and 95% confidence intervals were calculated using Der-Simonian and Laird method using random effects meta-analysis, taking into account conceptual heterogeneity. Odds ratios, hazard ratios and risk ratios were meta-analysed together and the results expressed as risk ratios, as the outcomes of interest are rare events. Given that included studies exhibited highly heterogenous calcium intake values, stratified meta-analysis was performed by average calcium intake, using 700 mg/day as threshold. Stratified meta-analyses were also performed by sex.

## Results

### Included participants

Supplementary Figure 1 details the participant population flowchart. A total of 17,968 men and women from the EPIC-Norfolk study were included in the analysis after the exclusion of participants with missing data on key variables and those with prevalent cancer or cardiovascular disease (myocardial infarction or stroke) at baseline. The mean age (SD) was 58.8 (9.2) years and there were 44.1% men. Patients were followed up for median (95%CI) of 7435 (7421–7442) days–20.36 (20.32–20.38) years. Total follow-up was 119,909,424 person-years. The characteristics of included and excluded participants are shown in Supplementary Table 4.

### Dietary calcium intake, mortality and cardiovascular disease

Table 1 displays the characteristics of participants divided by quintiles of calcium intake: calcium intake less than 770 mg/day was considered as the first quintile, whilst intakes between 771 and 926 mg/day, 927 and 1073 mg/day, 1074 and 1254 mg/day and more than 1254 mg/day represented the second, third, fourth and fifth quintiles respectively. Patients with a higher dietary calcium intake were predominantly male, had a lower body mass index (BMI), lower cholesterol levels, higher educational level, higher physical activity levels, lower levels alcohol consumption and were in greater proportion never smokers. Furthermore, participants with higher dietary calcium intake had higher total daily energy intake, fruit, vegetable and Vitamin D intake. Furthermore, they also had increased vitamin and calcium supplement use.

**Table 1:** Sample characteristics of 17,968 men and women of the EPIC-Norfolk cohort at study baseline (1997–2000) by quintiles of calcium intake

	Calcium intake < 770 mg	Calcium intake 771–926 mg	Calcium intake 927–1073 mg	Calcium intake 1074–1254 mg	Calcium intake ≥ 1255 mg	*P* value
	3594	3594	3593	3594	3593	
Age, mean (SD)	58.43 (9.18)	58.85 (9.11)	59.21 (9.32)	59.32 (9.34)	58.41 (9.09)	**< 0.001**
Females, N (%)	2185 (60.8)	2118 (58.932)	2039 (56.749)	1973 (54.897)	1729 (48.121)	**< 0.001**
Body mass index, mean (SD)	26.24 (3.90)	26.14 (3.78)	26.13 (3.73)	26.09 (3.71)	25.89 (3.62)	**0.001**
*Food frequency questionnaire, daily intake*						
Total energy intake (kJ), mean (SD)	6533.92 (1612.44)	7674.60 (1666.97)	8534.76 (1803.94)	9368.13 (2056.31)	11,144.41 (2535.38)	**< 0.001**
Fruit Intake (g), median (IQR)	170.00 (94.15–274.55)	201.55 (119.70–301.20)	212.25 (132.90–310.80)	222.48 (141.45–334.15)	250.00 (160.20–375.70)	**< 0.001**
Vegetable intake (g), mean (SD)	226.53 (110.31)	251.74 (114.23)	267.21 (117.50)	282.62 (122.74)	325.34 (167.53)	**< 0.001**
Alcohol intake (g), median (IQR)	4.68 (0.76–11.85)	4.68 (0.92–10.88)	4.59 (0.76–10.64)	3.62 (0.76–10.18)	4.03 (0.76–10.52)	**< 0.001**
Calcium intake (mg), mean (SD)	633.94 (110.22)	853.98 (45.11)	999.91 (42.61)	1159.35 (51.66)	1447.12 (185.02)	**< 0.001**
Vitamin D (µg), mean (SD)	2.35 (1.64–3.27)	2.81 (2.05–3.93)	3.11 (2.27–4.43)	3.40 (2.46–4.83)	4.12 (2.89–5.94)	**< 0.001**
Calcium supplements, N (%)	1389 (38.65)	1484 (41.291)	1463 (40.718)	1536 (42.738)	1569 (43.668)	**< 0.001**
Vitamin supplements, N (%)	1470 (40.9)	1537 (42.766)	1537 (42.778)	1578 (43.907)	1596 (44.42)	**0.029**
Hormone replacement therapy, used now or in the past, N (%)	751 (20.9)	686 (19.087)	658 (18.313)	620 (17.251)	546 (15.196)	**< 0.001**
Systolic blood pressure—mmHg, mean (SD)	134.81 (18.48)	134.80 (18.44)	135.29 (17.82)	134.87 (18.32)	134.73 (17.71)	0.700
Total cholesterol levels, mean (SD)	6.25 (1.19)	6.19 (1.14)	6.16 (1.14)	6.11 (1.12)	6.02 (1.10)	**< 0.001**
*Smoking status, N (%)*						**< 0.001**
Current smoker	477 (13.27)	411 (11.436)	361 (10.047)	351 (9.766)	338 (9.407)	
Former smoker	1493 (41.54)	1513 (42.098)	1477 (41.108)	1435 (39.928)	1463 (40.718)	
Never smoker	1624 (45.19)	1670 (46.466)	1755 (48.845)	1808 (50.306)	1792 (49.875)	
*Social status, N (%)*						0.406
Professional	226 (6.29)	239 (6.65)	265 (7.375)	263 (7.32)	268 (7.46)	
Manager	1343 (37.37)	1291 (35.921)	1312 (36.515)	1334 (37.117)	1302 (36.237)	
Skilled non-manual	590 (16.42)	610 (16.973)	609 (16.95)	640 (17.807)	571 (15.892)	
Skilled manual	820 (22.82)	828 (23.038)	818 (22.766)	820 (22.816)	838 (23.323)	
Semi-skilled	494 (13.75)	497 (13.829)	466 (12.97)	423 (11.77)	492 (13.693)	
Non-skilled	121 (3.37)	129 (3.589)	123 (3.423)	114 (3.172)	122 (3.395)	
*Education level, N (%)*						**0.019**
No qualification	2780 (77.35)	2766 (76.962)	2771 (77.122)	2729 (75.932)	2698 (75.09)	
O-level	374 (10.41)	397 (11.046)	374 (10.409)	378 (10.518)	363 (10.103)	
Higher degree	440 (12.24)	431 (11.992)	448 (12.469)	487 (13.55)	532 (14.807)	
*Physical activity, N (%)*						**< 0.001**
Inactive	1125 (31.3)	1044 (29.048)	1037 (28.862)	1023 (28.464)	828 (23.045)	
Moderately Inactive	1062 (29.55)	1102 (30.662)	1062 (29.557)	1015 (28.242)	1018 (28.333)	
Moderately active	829 (23.07)	829 (23.066)	792 (22.043)	887 (24.68)	880 (24.492)	
Active	578 (16.08)	619 (17.223)	702 (19.538)	669 (18.614)	867 (24.13)	
*Prevalent comorbidities at baseline, N (%)*						
Diabetes	58 (1.61)	73 (2.031)	63 (1.753)	72 (2.003)	61 (1.698)	0.589
Hypertension	459 (12.77)	479 (13.328)	486 (13.526)	481 (13.383)	439 (12.218)	0.441
*Medications at baseline, N (%)*						
Aspirin	171 (4.76)	180 (5.008)	199 (5.539)	193 (5.37)	178 (4.954)	0.555
Beta blockers	203 (5.65)	184 (5.12)	195 (5.427)	210 (5.843)	174 (4.843)	0.336
ACE inhibitors	0 (0)	2 (.056)	5 (.139)	0 (0)	2 (.056)	0.053
*Outcomes, N (%)*						
Incident all cardiovascular disease	1817 (50.56)	1875 (52.17)	1926 (53.604)	1896 (52.755)	1873 (52.129)	0.127
Incident acute myocardial infarction	196 (5.45)	215 (5.982)	196 (5.455)	233 (6.48)	193 (5.372)	0.197
Incident cerebrovascular disease	356 (9.91)	323 (8.987)	332 (9.24)	332 (9.238)	366 (10.186)	0.365
Incident cardiac failure	316 (8.79)	332 (9.238)	307 (8.544)	353 (9.82)	311 (8.66)	0.306
Incident aortic stenosis	80 (2.23)	72 (2.003)	88 (2.449)	80 (2.226)	82 (2.282)	0.795
Incident peripheral vascular disease	269 (7.48)	260 (7.234)	268 (7.46)	258 (7.179)	274 (7.626)	0.948
Incident all-cause mortality	1006 (27.99)	982 (27.323)	1058 (29.446)	1007 (28.019)	997 (27.748)	0.337
Incident cardiovascular disease mortality	321 (8.93)	274 (7.62)	328 (9.129)	314 (8.737)	303 (8.433)	0.174

Bold values indicate the statistically significant results (P < 0.05)

*SD* Standard deviation, *IQR* Inter-quartile range, *ACE* Angiotensin converting enzyme

Table 2 details the results of the multivariable Cox regression models. Compared to the first quintile of calcium intake (< 770 mg/day), the second (771–926 mg/day) and fourth (1074–1254 mg/day) quintiles of calcium intake were associated with lower all-cause mortality, respective HR (95%CI): 0.91 (0.83–0.99) and 0.85 (0.77–0.93). There were no associations between the third (1074–1254 mg/day) and fifth (≥ 1255 mg/day) quintiles of calcium intake and all-cause mortality, respective HR (95%CI): 0.95 (0.87–1.04) and 0.93 (0.83–1.04). Similarly, compared to the first quintile of calcium intake (< 770 mg/day), the second (771–926 mg/day) and fourth (1074–1254 mg/day) quintiles were associated with decreased cardiovascular mortality: HR (95%CI): 0.79 (0.67–0.93) and 0.80 (0.67–0.95) respectively. There were no associations between the third (1074–1254 mg/day) and fifth (≥ 1255 mg/day) quintiles of calcium intake and cardiovascular mortality, respective HR (95%CI): 0.90 (0.76–1.06) and 0.87 (0.71–1.07). Furthermore, compared to the first quintile of calcium intake (< 770 mg/day), the second (771–926 mg/day), third (927–1073 mg/day) and fourth (1074–1254 mg/day) quintiles were associated with a decreased rate of incident stroke, respective HR (95%CI): 0.84 (0.72–0.97), 0.83 (0.71–0.97) and 0.78 (0.66–0.92). There was no association between the fifth quintile (≥ 1255 mg/day) of calcium intake and incident stroke: 0.95 (0.78–1.15). There were no associations between calcium intake and incident cardiovascular disease, incident aortic stenosis, incident cardiac failure, incident myocardial infarction or incident peripheral vascular disease.

**Table 2:** Results of Cox regressions assessing the relationship between quintiles of calcium intake and incident mortality and cardiovascular events in 17,968 men and women of the EPIC-Norfolk study

Outcome	Calcium intake < 770 mg/day		Calcium intake 771–926 mg/day		Calcium intake 27–1073 mg/day		Calcium intake 1074–1254 mg/day		Calcium intake ≥ 1255 mg/day	
	HR (95% CI)	*P value*	HR (95% CI)	*P value*	HR (95% CI)	*P value*	HR (95% CI)	*P value*	HR (95% CI)	*P value*
*All*-*cause mortality*										
Unadjusted	1.00 (ref)		0.98 (0.90–1.07)	0.609	1.06 (0.98–1.16)	0.159	1.00 (0.91–1.09)	0.942	0.99 (0.91–1.08)	0.825
Fully adjusted	1.00 (ref)		**0.91 (0.83**–**0.99)**	**0.032**	0.95 (0.87–1.04)	0.291	**0.85 (0.77**–**0.93)**	**0.001**	0.93 (0.83–1.04)	0.207
*Cardiovascular mortality*										
Unadjusted	1.00 (ref)		0.85 (0.73–1.00)	0.056	1.03 (0.89–1.20)	0.680	0.97 (0.83–1.14)	0.741	0.94 (0.81–1.10)	0.463
Fully adjusted	1.00 (ref)		**0.79 (0.67**–**0.93)**	**0.005**	0.90 (0.76–1.06)	0.203	**0.80 (0.67**–**0.95)**	**0.012**	0.87 (0.71–1.07)	0.187
*Incident all cardiovascular disease*										
Unadjusted	1.00 (ref)		1.04 (0.98–1.11)	0.221	1.07 (1.01–1.14)	0.031	1.04 (0.98–1.11)	0.203	1.03 (0.96–1.10)	0.399
Fully adjusted	1.00 (ref)		0.99 (0.92–1.05)	0.704	0.99 (0.93–1.06)	0.817	0.94 (0.88–1.01)	0.102	1.02 (0.94–1.11)	0.601
*Incident aortic stenosis*										
Unadjusted	1.00 (ref)		0.90 (0.66–1.24)	0.520	1.12 (0.82–1.51)	0.476	1.00 (0.73–1.36)	0.978	1.02 (0.75–1.39)	0.878
Fully adjusted	1.00 (ref)		0.86 (0.62–1.19)	0.350	1.02 (0.74–1.41)	0.907	0.87 (0.62–1.24)	0.445	0.96 (0.64–1.43)	0.832
*Incident cardiac failure*										
Unadjusted	1.00 (ref)		1.06 (0.90–1.23)	0.495	0.98 (0.84–1.15)	0.813	1.11 (0.96–1.30)	0.163	0.98 (0.84–1.15)	0.805
Fully adjusted	1.00 (ref)		0.99 (0.85–1.16)	0.906	0.90 (0.76–1.07)	0.223	0.98 (0.83–1.17)	0.854	1.00 (0.82–1.22)	0.981
*Incident myocardial infarction*										
Unadjusted	1.00 (ref)		1.10 (0.90–1.33)	0.343	1.01 (0.83–1.23)	0.936	1.19 (0.98–1.44)	0.074	0.98 (0.81–1.20)	0.872
Fully adjusted	1.00 (ref)		1.04 (0.86–1.27)	0.668	0.93 (0.75–1.15)	0.491	1.06 (0.85–1.31)	0.610	0.92 (0.71–1.18)	0.492
*Incident peripheral arterial disease*										
Unadjusted	1.00 (ref)		0.96 (0.81–1.14)	0.680	1.01 (0.85–1.19)	0.931	0.95 (0.80–1.13)	0.566	1.02 (0.86–1.20)	0.841
Fully adjusted	1.00 (ref)		0.93 (0.79–1.11)	0.449	0.98 (0.82–1.17)	0.802	0.93 (0.77–1.12)	0.441	1.08 (0.87–1.34)	0.506
*Incident stroke*										
Unadjusted	1.00 (ref)		0.90 (0.78–1.05)	0.189	0.94 (0.81–1.09)	0.405	0.93 (0.80–1.08)	0.321	1.03 (0.89–1.19)	0.721
Fully adjusted	1.00 (ref)		**0.84 (0.72**–**0.97)**	**0.022**	**0.83 (0.71**–**0.97)**	**0.021**	**0.78 (0.66**–**0.92)**	**0.003**	0.95 (0.78–1.15)	0.584

Bold values indicate the statistically significant results (P < 0.05)

Adjusted for age, sex, body mass index, systolic blood pressure, low-density lipoprotein, high-density lipoprotein and total cholesterol levels, social class, education level, physical activity, alcohol intake, smoking status, pre-existing comorbidities (hypertension, stroke, myocardial infarction, diabetes), medication and supplement use (aspirin, statins, ACE inhibitors, beta-blockers, angiotensin receptor blockers, vitamin supplement use), dietary intake (total energy intake, fruit and vegetable, vitamin D and calcium intake) and current/former usage of hormone replacement therapy (HRT)

HR - hazard ratio; 95% CI - 95% confidence interval

### Supplemental calcium supplement, mortality and cardiovascular disease

Calcium supplement use was more common among participants who were older and female as well as those who had lower BMI, higher social status and higher education level (Supplementary Table 5). They were also more likely to have a higher level of physical activity, consume less alcohol and were in greater proportion never smokers. Calcium supplement use was associated with higher dietary calcium intake and increased vitamin supplement use. Calcium supplement users had a lower incidence of myocardial infarction, and peripheral vascular disease compared to those who did not use calcium supplements. Upon multivariable analysis, there was no significant association between calcium supplement use and any outcome (Table 3).

**Table 3:** Results of Cox regression assessing the relationship between calcium incident use and incident mortality and cardiovascular events in 17,968 men and women of the EPIC-Norfolk study

Outcome	HR (95% CI)		*P* value
	No calcium supplement	Calcium supplement use	
*Cardiovascular mortality*			
Unadjusted	1.00 (ref)	1.03 (0.97–1.08)	0.380
Fully adjusted	1.00 (ref)	1.01 (0.93–1.10)	0.825
*All*-*cause mortality*			
Unadjusted	1.00 (ref)	1.01 (0.92–1.12)	0.806
Fully adjusted	1.00 (ref)	0.88 (0.76–1.03)	0.103
*Incident all cardiovascular disease*			
Unadjusted	1.00 (ref)	1.01 (0.97–1.06)	0.492
Fully adjusted	1.00 (ref)	1.00 (0.94–1.06)	0.919
*Incident aortic stenosis*			
Unadjusted	1.00 (ref)	1.04 (0.86–1.27)	0.681
Fully adjusted	1.00 (ref)	1.01 (0.75–1.35)	0.969
*Incident cardiac failure*			
Unadjusted	1.00 (ref)	0.97 (0.88–1.07)	0.563
Fully adjusted	1.00 (ref)	0.92 (0.80–1.06)	0.255
*Incident myocardial infarction*			
Unadjusted	1.00 (ref)	0.87 (0.77–0.99)	0.034
Fully adjusted	1.00 (ref)	0.95 (0.79–1.15)	0.606
*Incident peripheral arterial disease*			
Unadjusted	1.00 (ref)	0.90 (0.81–1.01)	0.065
Fully adjusted	1.00 (ref)	1.01 (0.86–1.19)	0.927
*Incident stroke*			
Unadjusted	1.00 (ref)	0.98 (0.89–1.08)	0.702
Fully adjusted	1.00 (ref)	0.96 (0.83–1.10)	0.559

Adjusted for age, sex, body mass index, systolic blood pressure, low-density lipoprotein, high-density lipoprotein and total cholesterol levels, social class, education level, physical activity, alcohol intake, smoking status, pre-existing comorbidities (hypertension, stroke, myocardial infarction, diabetes), medication and supplement use (aspirin, statins, ACE inhibitors, beta-blockers, angiotensin receptor blockers, vitamin supplement use), dietary intake (total energy intake, fruit and vegetable, vitamin D and calcium intake) and current/former usage of hormone replacement therapy (HRT)

HR - hazard ratio; 95% CI - 95% confidence interval

### Secondary Analyses

There were several significant sex differences in the association between calcium intake and cardiovascular mortality and incident stroke (Supplementary Table 6). While amongst men, higher daily calcium intake (≥ 771 mg/day) was associated with 26–40% lower risk of cardiovascular mortality compared to the lowest calcium intake quintile (≤ 770 mg/day), there were no statistically significant associations between calcium intake and cardiovascular mortality amongst women. Similarly, while amongst men, the second (771–926 mg/day), third (927–2073 mg/day), and fourth (1074–1254 mg/day) quintiles of daily calcium intake were associated with 14–24% decreased risk of incident stroke compared to the lowest quintile of calcium intake (≤ 700 mg/day), there were no statistically significant relationship between calcium intake and the risk of stroke in women. Nevertheless, a further secondary analysis including only female participants having ever used HRT at baseline (n = 3261) revealed that in this population, compared to the lowest quintile of calcium intake (≤ 770 mg/day), the second (771–926 mg/day), third (927–1073 mg/day) and fourth (1074–1254 mg/day) quintiles were associated with a 27–30% lower risk of all-cause mortality (Supplementary Table 7). Furthermore, compared to the lowest quintile of calcium intake (≤ 770 mg/day), the highest quintile of calcium intake (≥ 1255 mg/day) was associated with a 75% decrease in the risk of incident aortic stenosis in this population, HR(95%CI): 0.25(0.06–0.95).

A secondary analysis restricting the analysed participant population to those without prevalent hypertension or diabetes mellitus at baseline (n = 15,396) revealed that the associations between calcium intake and all-cause and cardiovascular mortality were not preserved (Supplementary Table 8). Nevertheless, compared to the first quintile of calcium intake (< 770 mg/day), the second (771–926 mg/day), third (927–1073 mg/day) and fourth (1074–1254 mg/day) quintiles were associated with a decreased rate of incident stroke, respective HR (95%CI): 0.78 (0.66–0.94), 0.81 (0.67–0.97) and 0.75 (0.62–0.91). There was no association between the fifth quintile (≥ 1255 mg/day) of calcium intake and incident stroke in this population either.

Finally, RCS analyses modelling the non-linear relationship between calcium intake as a continuous variable and the pre-specified outcomes revealed similar results to the main analysis (Supplementary Figure 2). Compared to the mean daily calcium intake (1018.83 mg/day), lower calcium intake values were associated with significantly lower risk of all-cause mortality while there were no associations between higher-than-mean values of calcium intake and all-cause mortality. There were no associations between calcium intake as a continuous variable and cardiovascular mortality, incident all-cardiovascular disease, aortic stenosis, cardiac failure, myocardial infarction or peripheral arterial disease. Compared to the mean daily calcium intake (1018.83 mg/day), both very low (≤ 638 mg/day) and very high (≥ 1514 mg/day) daily calcium intake values were associated with significantly increased risk of incident stroke.

### Meta-analysis of prospective cohort studies including EPIC-Norfolk study

**Fig. 2:**
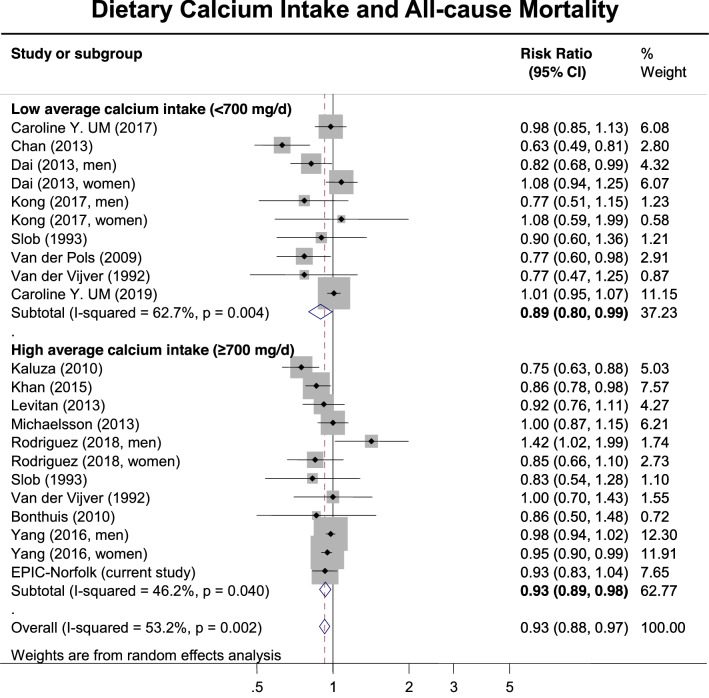
Meta-analysis (forest plot) of the association between dietary calcium intake (highest compared to the lowest level, mg/day) and risk of all-cause mortality stratified by low/high average (median) calcium intake among prospective cohort studies and the EPIC-Norfolk study. The diamond represents the summary risk ratio (pooled RR) estimate and its width shows corresponding 95% CI with random effects estimate. The size of the square and its central point reflect the study specific statistical weight (inverse of variance) and point estimate of the risk ratio respectively. The horizontal line reflects corresponding 95% CI. *I*^2^ test and Cochran’s Q statistic were used to assess statistical heterogeneity (*P *< 0.10) across studies. Odds ratios, hazard ratios and risk ratios from the primary studies were pooled together and the results expressed as risk ratios, as the outcomes of interest are rare events

**Fig. 3:**
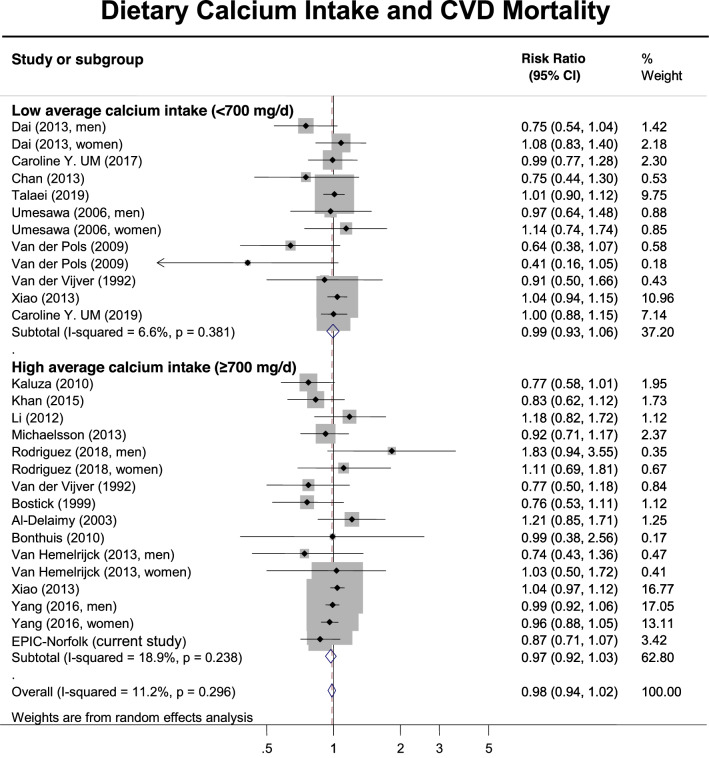
Meta-analysis (forest plot) of the association between dietary calcium intake (highest compared to the lowest level, mg/day) and risk of cardiovascular (CVD) mortality stratified by low/high average (median) calcium intake among prospective cohort studies and the EPIC-Norfolk study. The diamond represents the summary risk ratio (pooled RR) estimate and its width shows corresponding 95% CI with random effects estimate. The size of the square and its central point reflect the study specific statistical weight (inverse of variance) and point estimate of the risk ratio respectively. The horizontal line reflects corresponding 95% CI. *I*^2^ test and Cochran’s Q statistic were used to assess statistical heterogeneity (*P *< 0.10) across studies. Odds ratios, hazard ratios and risk ratios from the primary studies were pooled together and the results expressed as risk ratios, as the outcomes of interest are rare events

**Fig. 4:**
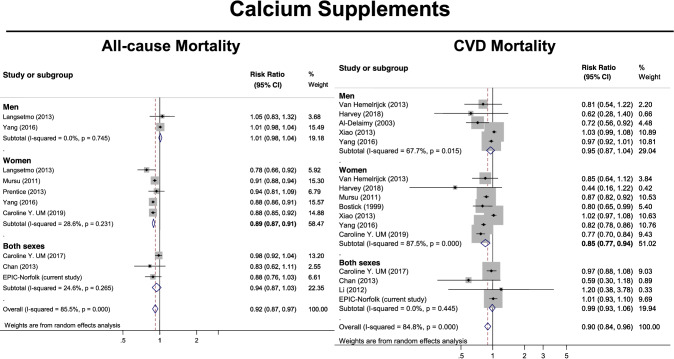
Meta-analysis (forest plot) of the association between calcium supplements (use versus non-use) and risk of all-cause and cardiovascular mortality stratified by sex among prospective cohort studies and EPIC-Norfolk study. The diamond represents the summary risk ratio (pooled RR) estimate and its width shows corresponding 95% CI with random effects estimate. The *I*^2^ test and Cochran’s Q statistic were used to assess statistical heterogeneity (*P *< 0.10) across studies. Odds ratios, hazard ratios and risk ratios from the primary studies were pooled together and the results expressed as risk ratios, as the outcomes of interest are rare events

Supplementary Table 3 shows the details of the studies included in the meta-analysis. The results of the meta-analyses are displayed in Figs. 2, 3 and 4. Figure 2 details the pooled results of studies evaluating the association between calcium intake and all-cause mortality, stratified by average calcium intake (low versus high). We included the risk ratios between the highest and lowest category of calcium intake, as defined by each individual study (Supplementary Table 3). Amongst studies with a low average calcium intake (< 700 mg/day), we identified an association between higher calcium intake and a decreased all-cause mortality risk, RR (95%CI) = 0.89 (0.80–0.99). This association was also identified amongst studies with a high average calcium intake (≥ 700 mg/day), RR (95%CI) = 0.93 (0.89–0.98). Supplementary Figure 3 details the pooled results of studies evaluating the association between calcium dietary intake and all-cause mortality, stratified by average calcium intake and sex. Amongst men, higher calcium intake was associated with decreased all-cause mortality in studies with low average calcium intake [0.82 (0.70–0.98)], but not in those with high average calcium intake [0.94 (0.82–1.09)]. Conversely, amongst women, higher dietary calcium intake was associated with decreased all-cause mortality in studies with high average calcium intake [0.95 (0.91-0.99)], but not in those with low average calcium intake [1.02 (0.96-1.07)].

Figure 3 details the pooled results of studies evaluating the association between calcium intake and cardiovascular mortality, stratified by average calcium intake (low versus high). We included the risk ratios between the highest and lowest category of calcium intake, as defined by each individual study (Supplementary Table 3). There was no association between calcium intake and cardiovascular mortality amongst either studies with low average calcium intake [0.99 (0.93–1.06)] or those with high average calcium intake [0.98 (0.94–1.02)]. Supplementary Figure 4 details the pooled results of studies evaluating the association between calcium dietary intake and all-cause mortality, stratified by average calcium intake and sex. Similarly, there were no associations between dietary calcium intake and cardiovascular mortality amongst either men or women from studies with either low or high average dietary calcium intake.

Figure 4 details the pooled results of the studies evaluating the association between calcium supplementation (use versus non-use) and mortality (cardiovascular and all-cause). Whilst calcium supplementation was associated with decreased all-cause mortality amongst women [0.89 (0.87–0.91)], there was no association between calcium supplementation and all-cause mortality amongst men [1.01 (0.98–1.04)]. Similarly, calcium supplementation was associated with decreased cardiovascular mortality amongst women [0.85 (0.77–0.94)], while there was no association between calcium supplementation and cardiovascular mortality amongst men [0.96 (0.87–1.04)].

## Discussion

The results of our prospective cohort study with > 20 years of follow-up suggest that, compared to a dietary calcium intake below 770 mg/day, intakes between 771–926 mg/day and 1074–1254 mg/day may be protective against both all-cause and cardiovascular mortality. We have also shown that, when compared to intakes below 770 mg/day, higher dietary calcium intakes below 1255 mg/day may be protective against incident stroke. Furthermore, we found no association between calcium intake and incident all-cardiovascular disease, as well as incident aortic stenosis, cardiac failure, myocardial infarction or peripheral vascular disease. Thus, calcium intake exhibited a ‘U’-shaped relationship with the risk of incident stroke with very low and very high intake values being associated with a higher risk. Our analyses also revealed several sex differences, in which the associations between calcium intake and incident adverse outcomes were only exhibited amongst men but not women. Another important subgroup to consider are women using HRT, in whom we found a ‘U’-shaped relationship between calcium intake and all-cause mortality, with very low and very high intake values being associated with a higher risk. Finally, we found no association between calcium supplement use and cardiovascular or all-cause mortality or incident cardiovascular disease. The results of our meta-analysis showed that higher dietary calcium intake was associated with lower all-cause mortality, but not cardiovascular mortality. Sex-stratified analyses revealed that amongst men, the association between higher dietary calcium intake and all-cause mortality was mainly driven by studies with low average calcium intake, while amongst women this association was driven by studies with a high average calcium intake. Finally, our meta-analysis also revealed that calcium supplementation was associated with decreased all-cause and cardiovascular mortality amongst women, but not men.

Previous systematic reviews have yielded highly heterogenous results, with some suggesting that an increased intake of calcium may be associated with an increased risk of cardiovascular disease [12, 13] and mortality, whilst others found no association [15, 16]. A further review, despite having included nine studies that found no association between increased dietary calcium intake and cardiovascular mortality, has found a U-shaped relationship between calcium intake and cardiovascular mortality [14]. Thus, the highest and the lowest consumers of calcium were at the greatest risk. Furthermore, a threshold effect was reported for the association between calcium intake and all-cause mortality: intakes lower than 900 mg/day were associated with a higher mortality risk, whilst increases in intake beyond 900 mg/day were not associated with any further risk decreases. The latter finding may potentially account for the lack of overall effect in our analysis, given that in our database cohort 900 mg/day represents approximately the 36th centile. Similarly, a previous meta-analysis has found that whilst calcium intakes ranging between 200 and 1500 mg/day were not associated with incident cardiovascular disease, but found an association between calcium supplementation and incident myocardial infarction [16]. One of the most striking results of our database analysis was that stroke incidence may be reduced in association with an increased dietary calcium intake, but only up to 1255 mg/day. This may be potentially explained through the intermediate antihypertensive properties of calcium [7], which is a major risk factor for stroke. Nevertheless, the fact that intakes above 1255 mg/day cease to be protective against incident stroke may be attributed to the association between serum calcium levels and carotid artery plaque thickness [8].

Our study benefits from several strengths. Our database study was based on a large prospective cohort with a very long follow-up (> 20 years). Furthermore, our study cohort included both males and females allowing for a more comprehensive analysis than those studies focussing solely on men [25] or women [2, 5, 30]. We were also able to adjust for a wide range of potential confounders including socio-demographic, lifestyle, co-morbidity, medication and supplement use data.

Nevertheless, a few limitations of our study should be considered. Firstly, as a prospective cohort study, calcium exposure was not randomly assigned, raising the possibility of residual confounding. Furthermore, given the lack of time-updated data on participant characteristics, we only adjusted our time-to-event analyses with a long follow-up time for participant characteristics at baseline. Additionally, dietary intakes were calculated from food frequency questionnaires (FFQ), which may be prone to recall bias. However, the FFQ used in the EPIC-Norfolk study has been validated and required participants to consider their average dietary intake over a long period of time, covering a longer duration than methods such as the seven-day dietary diary or 24-h dietary recall [45]. Our study also analysed calcium intake regardless of dietary source and we were thus unable to draw conclusions dependent of the dietary source of calcium. Finally, our study data did not allow for consideration of the water intake of individual participants. Nevertheless, a previous study found no association between high water hardness and incident coronary heart disease in a British cohort [46].

In conclusion, our results demonstrate that moderate dietary calcium intake may be beneficial against cardiovascular and all-cause mortality as well as incident stroke in the general adult population, compared to low and high intakes. Our results suggest that these associations are more likely to be observed amongst men as well as women having received HRT. Calcium supplementation use was not associated with increased mortality or cardiovascular risk in our population. Nevertheless, calcium supplementation may be associated with decreased all-cause and cardiovascular mortality amongst women based on our meta-analysis results. The clinical implications of our study in the UK setting may be that those with risk factors for stroke should be encouraged to meet the UK dietary calcium recommendations of ≥ 700 mg/day, but not exceed a higher limit of 1255 mg of calcium per day. Additionally, a moderate intake of dietary calcium (771–926 mg/day) [47] may be a protective factor against mortality. Calcium supplementation may also be actively encouraged amongst women. More research is needed to establish a clear association between calcium intake and incident stroke and other cardiovascular disease, with particular emphasis on determining whether an upper threshold of dietary calcium intake for this relationship does indeed exist.

## Electronic supplementary material

Below is the link to the electronic supplementary material.

Supplementary material 1 (DOCX 1404 kb)

## Data Availability

The data that support the findings of this study are available from the corresponding author, upon reasonable request.

## Code availability

The code utilised to complete the statistical analysis of this study is available from the corresponding author, upon reasonable request.
