# Supplementary material for: Calcium intake, calcium supplementation and cardiovascular disease and mortality in the British population: EPIC-norfolk prospective cohort study and meta-analysis
Source: Eur J Epidemiol. 2020 Dec 31;36(7):669–83. doi: 10.1007/s10654-020-00710-8 (PMC8403619; doi:10.1007/s10654-020-00710-8)
Supplement: Supplementary file 1 — Supplementary material 1 (DOCX 1404 kb) [file 10654_2020_710_MOESM1_ESM.doc]

**Supplementary Material**

**Supplementary Table 1.** Best fitting models for each regression model assessing the relationship between calcium intake as a continuous variable and the risk of incident adverse outcomes.

| **Outcome** | **Best fitting model** |
| --- | --- |
| All-cause mortality | RCS – 2 df |
| Cardiovascular mortality | Linear |
| Incident cardiovascular disease | Linear |
| Incident aortic stenosis | Linear |
| Incident cardiac failure | Linear |
| Incident myocardial infarction | Linear |
| Incident peripheral arterial disease | Linear |
| Incident stroke | RCS – 2 df |

RCS – restricted cubic splines; df – degrees of freedom;

**Supplementary Table 2.** Description of cohort studies evaluating dietary calcium and/or calcium supplement and risk of cardiovascular events included in meta-analysis.

| Study ID | Design | Country | Participants | Age | Male% | Inclusion criteria |
| --- | --- | --- | --- | --- | --- | --- |
| Bonthuis, 2010 | Prospective cohort study | Australia | 1,529 | Mean 50 years | 43% | Participants were randomly selected adult residents of Nambour, Australia in a skin cancer prevention trial. |
| Chan, 2013 | Prospective cohort study | China | 3,139 | Mean 72 years | 48% | People living in community in Hong Kong aged ≥65 years. |
| Dai, 2013 | Prospective cohort study | China | 136,442 | 40-74 years | 45% | Participants were age 40-74 years in the Shanghai Women's Health Study and Shanghai Men's Health Study. |
| Kaluza, 2010 | Prospective cohort study | Sweden | 23,366 | 45-79 years | 100% | Participants were men in the Cohort of Swedish Men. |
| Langsetmo, 2013 | Prospective cohort study | Canada | 9,033 | 63 years | 30% | Participants were in the Canadian Multicentre Osteoporosis Study. |
| Li, 2012 | Prospective cohort study | Germany | 23,980 | 51 years | 46% | Participants were Germans age 35-64 years in EPIC Heidelberg cohort. |
| Michaelsson, 2013 | Prospective cohort study | Sweden | 61,433 | Born between 1914 and 1948 | 0% | Swedish women in Swedish Mammography cohort. |
| Mursu, 2011 | Prospective cohort study | USA | 38,772 | 62 years | 0% | Participants were older women age 55-69 years at baseline in the Iowa Women's Health Study. |
| Prentice, 2013 | Prospective cohort study | USA | 93,676 | Unclear | Unclear | Participants were women in the Women's Health Initiative Observational Study. |
| Umesawa, 2006 | Prospective cohort study | Japan | 110,792 | 40-79 years | 42% | Participants were Japanese aged 40-79 years in JACC study. |
| Van der Vijver, 1992 | Prospective cohort study | Netherlands | 2,605 | 52 years | 51% | Participants were Dutch civil servants who volunteered to participant in the study. |
| Van Hemelrijck, 2013 | Prospective cohort study | USA | 20,024 | 46 years | 47% | Participants were age 17 years and over in NHANES III study. |
| Xiao, 2013 | Prospective cohort study | USA | 388,229 | 62 years | 56% | Participants were in the National Institute of Health-AARP Diet and Health Study. |
| Caroline Y. Um, 2017 | Prospective cohort study | USA | 21,427 | 45 years and older | 44.1% | The REGARDS study, participants (black and white men and women) aged 45 years and older from the continental United States and blacks and persons residing in the Stroke Belt. |
| Khan, 2015 | Prospective cohort study | Australia | 41,514 | 40-69 years | 41% | Participants (men and women aged 40 to 69 years at baseline) were from the Melbourne Collaborative Cohort Study. |
| Kong, 2017 | Prospective cohort study | Republic of Korea | 4,311 | >50 years  59.2±5.8 y men 60.5±5.3 y women | 50% | 2158 men and 2153 women aged >50 y were recruited from among residents of Ansung and Ansan who lived in the survey area for >=6 months before enrollment beginning in 2001. |
| Al-Delaimy, 2003 | Prospective cohort study | USA | 39,800 | 40-79 years | 100% | 39,800 men aged 40–75 years from The Health Professionals Follow-up Study answered a detailed questionnaire by mail on diet  and medical history in 1986 were followed up for 12 y. |
| Bostick, 1999 | Prospective cohort study | USA | 34,486 | 55-69 years | 0% | 34,486 postmenopausal Iowa (The Iowa Women's Health Study cohort) women 55-69 years old and without a history of ischemic heart disease who completed a dietary questionnaire in 1986. |
| Levitan, 2013 | Prospective cohort study | USA | 3,340 | 50-79 years | 0% | Women’s Health Initiative, 3,340 women aged 50–79 years at the study entry, recruited between 1993 and 1998 through forty clinical centers and was closed in 2004–05. |
| Slob, 1993 | Prospective cohort study | Dutch,  Amsterdam | 2,591 | 40-65 years | 51% | General health examination in 1953-1954 among Dutch civil servants and their spouses aged 40 to 65 years (2591 participants) in a 28-year follow-up. |
| Van der Pols, 2009 | Prospective cohort study | UK and Scotland | 4,374 | 8 years (4-11) | 49% | 4999 children in England and Scotland (Boyd Orr cohort) participated in a study of family food consumption in 1937–9. Cause of death was ascertained between 1948 and 2005 in 4374 traced cohort members with complete data. |
| Rodriguez, 2018 | Prospective cohort study | Australia | 34,627 | 40-69 years | 39.8% | Melbourne Collaborative Cohort Study (MCCS) of community-dwelling Australian adults men and women aged 40–69 at baseline  (1990–1994). The sample was largely of Anglo-Saxon origin, but approximately 25% were born in Southern Europe. |
| Caroline Y. UM, 2019 | Prospective cohort study | USA | 35,221 | 55–69 years | 0% | 35,221 women randomly selected from 1985 Iowa driver’s license information (IWHS) returned completed questionnaires on demographics, medical history, lifestyle, and diet with follow-up surveys mailed in 1987, 1989, 1992, 1997, and 2004. |
| Yang, 2016 | Prospective cohort study | USA | 132,823 | Mean 62.6 years | 45% | A total of 132,823 adults men and women participants in the Cancer Prevention Study II (CPS-II) Nutrition Cohort were followed from baseline (1992 or 1993) through 2012. |
| Harvey, 2018 | Prospective cohort study | UK | 502,637 | 40-69 years | 44.2% | UK Biobank study, comprising 502,637 men and women aged 40 to 69 years at recruitment. Outcome data derived from National Health Service records. |
| Talaei, 2019 | Prospective cohort study | Singapore | 57,078 | 45-74 years | 46% | 57,078 participants of the Singapore Chinese Health Study (SCHS) aged 45 to 74 years with data at baseline (1993–1998) were recruited from Hokkiens and Cantonese, the 2 major dialect groups in Singapore. |

**Supplementary Table 3.** Assessment of dietary calcium intake or supplement, outcomes and results of cohort studies included in meta-analysis

| Study ID | Measure of dietary intake | Outcomes | Follow up | Results |
| --- | --- | --- | --- | --- |
| Bonthuis, 2010 | Self-administered, semi-quantitative food frequency questionnaire | All-cause mortality and CVD mortality | Average 14.4 years | Dietary calcium intake and all-cause mortality: tertile 3 vs 1 aHR 0.86 (0.50-1.48).  Dietary calcium intake and CVD mortality: tertile 3 vs 1 aHR 0.99 (0.38-2.56). |
| Chan, 2013 | Validated food frequency questionnaire | CVD mortality | Median 9.1 years | Calcium intake and all-cause mortality: quartile 4 vs 1 aHR 0.63 (0.49-0.81).  Calcium intake and CVD mortality: quartile 4 vs 1 aHR 0.75 (0.44-1.30).  Calcium supplement and all-cause mortality: aHR 0.83 (0.62-1.11).  Calcium supplement and CVD mortality: aHR 0.59 (0.30-1.18). |
| Dai, 2013 | Interviews using validated food-frequency questionnaire | All-cause mortality and CVD mortality | Up to 13 years | Calcium intake and all-cause mortality in women: tertile 3 vs 1 aHR 1.08 (0.94-1.25).  Calcium intake and all-cause mortality in men: tertile 3 vs 1 aHR 0.82 (0.68-0.99).  Calcium intake and CVD mortality in women: tertile 3 vs 1 aHR 1.08 (0.83-1.40).  Calcium intake and CVD in men: tertile 3 vs 1 aHR 0.75 (0.54-1.04). |
| Kaluza, 2010 | Food-frequency questionnaire | CVD mortality | Average 10 years | Calcium intake and all-cause mortality: >1,599 mg/day vs <1,230 mg/day aHR 0.75 (0.63-0.88).  Calcium intake and CVD mortality: >1,599 mg/day vs <1,230 mg/day aHR 0.77 (0.58-1.01). |
| Langsetmo, 2013 | Semi-quantitative food-frequency questionnaire | All-cause mortality | 10 years | Calcium supplement and all-cause mortality in women: aHR 0.78 (0.66-0.92).  Calcium supplement and all-cause mortality in men: aHR 1.05 (0.83-1.32). |
| Li, 2012 | Food-frequency questionnaire | CVD mortality | Average 11 years | Dietary calcium and CVD mortality: quartile 4 vs 1 aHR 1.18 (0.82-1.72).  Calcium supplement and CVD mortality: aHR 1.20 (0.38-3.78). |
| Michaelsson, 2013 | Food-frequency questionnaire | All-cause and CVD mortality | Median 19 years | Dietary calcium groups and all-cause mortality: quartile 4 vs 1 aHR 1.00 (0.87-1.15).  Dietary calcium groups and CVD mortality: quartile 4 vs 1 aHR 0.92 (0.71-1.17). |
| Mursu, 2011 | Validated food-frequency questionnaire | All-cause mortality | Mean 19 years | Calcium supplement and all-cause mortality: aHR 0.91 (0.88-0.94).  Calcium supplement and CVD mortality: aHR 0.87 (0.82-0.92). |
| Prentice, 2013 | Patients randomized to calcium supplement. | All-cause mortality | Average 7 years | Calcium supplementation and all-cause mortality: 0.94 (0.81-1.09). |
| Umesawa, 2006 | Validated dietary questionnaire | CVD mortality | Average 9.6 years | Calcium intake and CVD mortality in men: quintile 5 vs 1 aRR 0.97 (0.64-1.48).  Calcium intake and CVD mortality in women: quintile 5 vs 1 aRR 1.14 (0.74-1.74). |
| Van der Vijver, 1992 | Survey with dietary intake through 1-week food frequency recall | All-cause and CVD mortality | 28 years | Calcium intake and all-cause mortality in men: quintile 5 vs 1 aOR 1.00 (0.70-1.43)  Calcium intake and CVD mortality in men: quintile 5 vs 1 aOR 0.77 (0.50-1.18)  Calcium intake and all-cause mortality in women: quintile 5 vs 1 aOR 0.77 (0.47-1.25)  Calcium intake and CVD mortality in women: quintile 5 vs 1 aOR 0.91 (0.50-1.66) |
| Van Hemelrijck, 2013 | Diet from 24 hour dietary recall which was validated | CVD mortality | Up to 18 years | Dietary calcium and CVD mortality in men: quartile 4 vs 1 aHR 0.74 (0.43-1.36)  Dietary calcium and CVD mortality in women: quartile 4 vs 1 aHR 1.03 (0.50-1.72)  Calcium supplement and CVD mortality in men: HR 0.81 (0.54–1.22)  Calcium supplement and CVD mortality in women: HR 0.85 (0.64–1.12) |
| Xiao, 2013 | Food-frequency questionnaire | CVD mortality | Average 12 years | Calcium intake and CVD mortality in men: quintile 5 vs 1 aRR 1.04 (0.97-1.12)  Calcium intake and CVD mortality in women: quintile 5 vs 1 aRR 1.04 (0.94-1.15).  Supplemental calcium and CVD mortality in men: aRR 1.03 (0.99-1.08)  Supplemental calcium and CVD mortality in women: aRR 1.02 (0.97-1.08) |
| Caroline Y. Um, 2017 | Self-administered Block food  frequency questionnaire | All-cause and CVD mortality | 10 years | Dietary calcium and all-cause mortality: quintile 5 vs 1 aHR 0.98 (0.85-1.13)  Dietary calcium and CVD mortality: quintile 5 vs 1 aHR 0.99 (0.77-1.28)  Supplemental calcium and all-cause mortality: quintile 5 vs 1 aHR 0.98 (0.92-1.04)  Supplemental calcium and CVD mortality: quintile 5 vs 1 aHR 0.97 (0.88-1.08) |
| Khan, 2015 | Food-frequency questionnaire | All-cause and CVD mortality | Mean 12 years | Dietary calcium intake and all-cause mortality: quartile 4 vs 1aHR 0.86 (0.76-0.98)  Dietary calcium intake and CVD mortality: quartile 4 vs 1 aHR 0.83 (0.62-1.12) |
| Kong, 2017 | Semi-quantitative, 103-item food frequency questionnaire | All-cause mortality | Median 9 years | Dietary calcium and all-cause mortality in men: quartile 4 vs 1aHR 0.77 (0.51-1.15)  Dietary calcium and all-cause mortality in women: quartile 4 vs 1aHR 1.08 (0.59-1.99) |
| Al-Delaimy, 2003 | 131-item semi-quantitative  food-frequency questionnaire | CVD mortality | 12 years | Dietary calcium and IHD mortality in men: quintile 5 vs 1 aHR 1.21 (0.85-1.71)  Calcium supplement and IHD mortality in men: aHR 0.72 (0.56-0.92) |
| Bostick, 1999 | Semi-quantitative food frequency questionnaire | CVD mortality | 8 years | Dietary calcium and IHD mortality in women: quartile 4 vs 1 aHR 0.76 (0.53-1.11)  Calcium supplement and IHD mortality in women: aHR 0.80 (0.65-0.99) |
| Levitan, 2013 | Food-frequency questionnaire | All-cause mortality | Median 4.6 years | Dietary calcium and all-cause mortality in women: quartile 4 vs 1 aHR 0.92 (0.76-1.11) |
| Slob, 1993 | Food-frequency questionnaire | All-cause mortality | 28 years | Dietary calcium and all-cause mortality in men: quintile 5 vs 1 aHR 0.90 (0.60-1.36)  Dietary calcium and all-cause mortality in women: quintile 5 vs 1 aHR 0.83 (0.54-1.28) |
| Van der Pols, 2009 | 7-day household inventory weighted food recall | All-cause and CVD mortality | 65 years | Dietary calcium and all-cause mortality: quartile 4 vs 1 aHR 0.77 (0.60-0.98)  Dietary calcium and CHD mortality: quartile 4 vs 1 aHR 0.64 (0.38-1.07)  Dietary calcium and stroke mortality: quartile 4 vs 1 aHR 0.41 (0.16-1.05) |
| Rodriguez, 2018 | Food-frequency questionnaire | All-cause and CVD mortality | 12.5 years | Dietary calcium and all-cause mortality in men: quartile 4 vs 1 aHR 1.42 (1.02-1.99)  Dietary calcium and all-cause mortality in women: quartile 4 vs 1 aHR 0.85 (0.66-1.10)  Dietary calcium and CVD mortality in men: quartile 4 vs 1 aHR 1.83 (0.94-3.55)  Dietary calcium and CVD mortality in women: quartile 4 vs 1 aHR 1.11 (0.69-1.81) |
| Caroline Y. UM, 2019 | self-administered, 127-food item Willett food frequency questionnaire | All-cause and CVD mortality | 25 years | Dietary calcium and all-cause mortality in women: quintile 5 vs 1 aHR 1.01 (0.95-1.07)  Dietary calcium and CHD mortality in women: quintile 5 vs 1 aHR 1.00 (0.88-1.15)  Calcium supplement and all-cause mortality in women: aHR 0.88 (0.85-0.92)  Calcium supplement and CHD mortality in women: aHR 0.77 (0.70-0.84) |
| Yang, 2016 | 68-item modiﬁed Block food-  frequency questionnaire | All-cause and CVD mortality | 17.5 years | Dietary calcium and all-cause mortality in men: quintile 5 vs 1 aHR 0.98 (0.94, 1.02)  Dietary calcium and all-cause mortality in women: quintile 5 vs 1 aHR 0.95 (0.90, 0.99)  Dietary calcium and CVD mortality in men: quintile 5 vs 1 aHR 0.99 (0.92, 1.06)  Dietary calcium and CVD mortality in women: quintile 5 vs 1 aHR 0.96 (0.88, 1.05)  Calcium supplement and all-cause mortality in men: quintile 5 vs 1 aHR 1.01 (0.98-1.04)  Calcium supplement and all-cause in women: quintile 5 vs 1 aHR 0.88 (0.86-0.91)  Calcium supplement and CVD mortality in men: quintile 5 vs 1 aHR 0.97 (0.92-1.01)  Calcium supplement and CVD mortality in women: quintile 5 vs 1 aHR 0.82 (0.78-0.86) |
| Harvey, 2018 | Touch screen questionnaire for calcium supplement. | CVD mortality | Average 7 years | Calcium supplement and IHD mortality in men: aHR 0.62 (0.28-1.40)  Calcium supplement and IHD mortality in women: aHR 0.44 (0.16-1.22) |
| Talaei, 2019 | Validated 165-item food frequency questionnaire | CVD mortality | 17.2 years | Dietary calcium and CVD mortality: quintile 5 vs 1 aHR 1.01 (0.90-1.12) |

**Supplementary Table 4.** Characteristics of participants included and excluded from analysis in EPIC-Norfolk cohort.

|  | **Excluded**  **(n=7671)** | **Included**  **(n=17,968)** | ***P* value** |
| --- | --- | --- | --- |
| Age, mean (SD) | 60.18 (9.49) | 58.84 (9.22) | 0.144 |
| Females, N (%) | 3988 (51.99) | 10044 (55.899) | <0.001 |
| Body Mass Index, mean (SD) | 26.99 (4.22) | 26.10 (3.75) | <0.001 |
| Food Frequency Questionnaire, daily intake |  |  |  |
| Total Energy Intake (kJ), mean (SD) | 8519.02 (2546.91) | 8651.03 (2508.60) | <0.001 |
| Fruit Intake (g), median (IQR) | 206.50 (120.15-323.80) | 211.60 (128.55-320.85) | 0.072 |
| Vegetable Intake (g), mean (SD) | 270.80 (141.51) | 270.68 (132.35) | <0.001 |
| Alcohol Intake (g), median (IQR) | 3.60 (0.76-10.88) | 4.66 (0.76-10.88) | 0.003 |
| Calcium Intake (mg), mean (SD) | 996.64 (301.59) | 1018.83 (293.85) | <0.001 |
| Vitamin D (µg), mean (SD) | 3.04 (2.10-4.50) | 3.09 (2.20-4.57) | 0.002 |
| Vitamin Supplements, N (%) | 4597 (59.93) | 7441 (41.41) | <0.001 |
| Hormone replacement therapy, used now or in the past, N (%) | 2949 (38.44) | 7718 (42.95) | <0.001 |
| Systolic Blood Pressure – mmHg, mean (SD) | 1145 (14.93) | 3261 (18.15) | <0.001 |
| Total Cholesterol levels, mean (SD) | 136.90 (19.08) | 134.90 (18.16) | <0.001 |
| Smoking Status, N (%) |  |  | <0.001 |
| Current Smoker | 1046 (13.64) | 1938 (10.786) |  |
| Former Smoker | 3380 (44.06) | 7381 (41.079) |  |
| Never Smoker | 3245 (42.3) | 8649 (48.136) |  |
| Social Status, N (%) |  |  | 0.097 |
| Professional | 493 (6.43) | 1261 (7.018) |  |
| Manager | 2576 (33.58) | 6582 (36.632) |  |
| Skilled non-manual | 1119 (14.59) | 3020 (16.808) |  |
| Skilled manual | 1648 (21.48) | 4124 (22.952) |  |
| Semi-skilled | 989 (12.89) | 2372 (13.201) |  |
| Non-skilled | 276 (3.6) | 609 (3.389) |  |
| Education Level, N (%) |  |  | 0.024 |
| No qualification | 5984 (78.01) | 13744 (76.492) |  |
| O-Level | 736 (9.59) | 1886 (10.496) |  |
| Higher Degree | 951 (12.4) | 2338 (13.012) |  |
| Physical Activity, N (%) |  |  | <0.001 |
| Inactive | 2806 (36.58) | 5057 (28.144) |  |
| Moderately Inactive | 2092 (27.27) | 5259 (29.269) |  |
| Moderately Active | 1559 (20.32) | 4217 (23.47) |  |
| Active | 1213 (15.81) | 3435 (19.117) |  |
| Prevalent comorbidities at baseline, N (%) |  |  |  |
| Diabetes | 261 (3.4) | 327 (1.82) | <0.001 |
| Hypertension | 1319 (17.19) | 2344 (13.05) | <0.001 |
| Medications at baseline, N (%) |  |  |  |
| Aspirin | 1017 (13.26) | 921 (5.13) | <0.001 |
| Beta blockers | 656 (8.551) | 966 (5.38) | <0.001 |
| ACE inhibitors | 2 (.03) | 9 (.05) | 0.395 |
| Outcomes, N (%) |  |  |  |
| Incident all cardiovascular disease | 4598 (59.94) | 9387 (52.24) | <0.001 |
| Incident acute myocardial infarction | 680 (8.86) | 1033 (5.75) | <0.001 |
| Incident cerebrovascular disease | 977 (12.74) | 1709 (9.51) | <0.001 |
| Incident cardiac failure | 1047 (13.65) | 1619 (9.01) | <0.001 |
| Incident aortic stenosis | 223 (2.91) | 402 (2.24) | 0.001 |
| Incident peripheral vascular disease | 830 (10.82) | 1329 (7.4) | <0.001 |
| Incident all-cause mortality | 2989 (38.96) | 5050 (28.11) | <0.001 |
| Incident cardiovascular disease mortality | 1077 (14.04) | 1540 (8.57) | <0.001 |

SD – standard deviation; IQR – inter-quartile range ; ACE – angiotensin converting enzyme

**Supplementary Table 5.** Sample characteristics of 17,967 men and women of the EPIC-Norfolk cohort at study baseline (1997-2000) by use of calcium supplement

|  | **No calcium supplements**  **(n=10527)** | **Calcium supplements**  **(n=7441)** | ***P* value** |
| --- | --- | --- | --- |
| Age, mean (SD) | 58.40 (9.25) | 59.47 (9.14) | <0.001 |
| Females, N (%) | 5237 (49.75) | 4807 (64.602) | <0.001 |
| Body Mass Index, mean (SD) | 26.28 (3.77) | 25.83 (3.72) | <0.001 |
| Food Frequency Questionnaire, daily intake |  |  |  |
| Total Energy Intake (kJ), mean (SD) | 8719.00 (2569.74) | 8554.88 (2416.36) | <0.001 |
| Fruit Intake (g), median (IQR) | 193.10 (114.20-295.90) | 239.05 (148.15-351.90) | <0.001 |
| Vegetable Intake (g), mean (SD) | 262.91 (129.20) | 281.68 (135.94) | <0.001 |
| Alcohol Intake (g), median (IQR) | 4.68 (0.76-10.89) | 4.61 (0.76-10.53) | 0.036 |
| Calcium Intake (mg), mean (SD) | 1011.36 (292.80) | 1029.41 (295.03) | <0.001 |
| Vitamin D (µg), mean (SD) | 3.08 (2.18-4.55) | 3.09 (2.21-4.60) | 0.373 |
| Vitamin Supplements, N (%) | 1340 (12.73) | 6378 (85.71) | <0.001 |
| Hormone replacement therapy, used now or in the past, N (%) | 1599 (15.19) | 1662 (22.34) | <0.001 |
| Systolic Blood Pressure – mmHg, mean (SD) | 135.04 (18.10) | 134.70 (18.23) | 0.224 |
| Total Cholesterol levels, mean (SD) | 6.13 (1.14) | 6.17 (1.14) | 0.027 |
| Smoking Status, N (%) |  |  | <0.001 |
| Current Smoker | 1320 (12.54) | 618 (8.305) |  |
| Former Smoker | 4318 (41.02) | 3063 (41.164) |  |
| Never Smoker | 4889 (46.44) | 3760 (50.531) |  |
| Social Status, N (%) |  |  | <0.001 |
| Professional | 758 (7.2) | 503 (6.76) |  |
| Manager | 3777 (35.88) | 2805 (37.697) |  |
| Skilled non-manual | 1657 (15.74) | 1363 (18.317) |  |
| Skilled manual | 2516 (23.9) | 1608 (21.61) |  |
| Semi-skilled | 1432 (13.6) | 940 (12.633) |  |
| Non-skilled | 387 (3.68) | 222 (2.983) |  |
| Education Level, N (%) |  |  | 0.014 |
| No qualification | 8004 (76.03) | 5740 (77.14) |  |
| O-Level | 1089 (10.34) | 797 (10.711) |  |
| Higher Degree | 1434 (13.62) | 904 (12.149) |  |
| Physical Activity, N (%) |  |  | <0.001 |
| Inactive | 3130 (29.73) | 1927 (25.897) |  |
| Moderately Inactive | 2941 (27.94) | 2318 (31.152) |  |
| Moderately Active | 2456 (23.33) | 1761 (23.666) |  |
| Active | 2000 (19) | 1435 (19.285) |  |
| Prevalent comorbidities at baseline, N (%) |  |  |  |
| Diabetes | 219 (2.08) | 108 (1.45) | 0.002 |
| Hypertension | 1364 (12.96) | 980 (13.17) | 0.676 |
| Medications at baseline, N (%) |  |  |  |
| Aspirin | 444 (4.22) | 477 (6.41) | <0.001 |
| Beta blockers | 575 (5.46) | 391 (5.25) | 0.544 |
| ACE inhibitors | 5 (.05) | 4 (.05) | 0.853 |
| Outcomes, N (%) |  |  |  |
| Incident all cardiovascular disease | 5497 (52.22) | 3890 (52.28) | 0.937 |
| Incident acute myocardial infarction | 640 (6.08) | 393 (5.28) | 0.024 |
| Incident cerebrovascular disease | 1014 (9.63) | 695 (9.34) | 0.511 |
| Incident cardiac failure | 965 (9.17) | 654 (8.79) | 0.384 |
| Incident aortic stenosis | 233 (2.21) | 169 (2.27) | 0.796 |
| Incident peripheral vascular disease | 815 (7.74) | 514 (6.91) | 0.035 |
| Incident all-cause mortality | 2945 (27.98) | 2105 (28.29) | 0.645 |
| Incident cardiovascular disease mortality | 902 (8.57) | 638 (8.57) | 0.989 |

SD – standard deviation; IQR – inter-quartile

**Supplementary Table 6.** Results of sex-stratified Cox regressions assessing the relationship between quintiles of calcium intake and incident mortality and cardiovascular events in 17,968 men and women of the EPIC-Norfolk study.

| **Outcome** | **Calcium intake**  **<770 mg/day** | | **Calcium intake**  **771-926 mg/day** | | **Calcium intake**  **927-1073 mg/day** | | **Calcium intake 1074-1254 mg/day** | | **Calcium intake ≥1255 mg/day** | |
| --- | --- | --- | --- | --- | --- | --- | --- | --- | --- | --- |
|  | **HR (95% CI)** | ***P (int)*** | **HR (95% CI)** | ***P (int)*** | **HR (95% CI)** | ***P (int)*** | **HR (95% CI)** | ***P (int)*** | **HR (95% CI)** | ***P (int)*** |
| All-cause mortality | | | | | | | | | | |
| Men | 1.00 (ref) |  | 0.84 (0.74-0.96) | 0.117 | 0.92 (0.81-1.04) | 0.455 | 0.82 (0.72-0.94) | 0.548 | 0.89 (0.77-1.02) | 0.276 |
| Women | 1.00 (ref) |  | 0.97 (0.86-1.10) |  | 0.98 (0.87-1.11) |  | 0.87 (0.76-0.99) |  | 0.98 (0.84-1.13) |  |
| Cardiovascular mortality | | | | | | | | | | |
| Men | 1.00 (ref) |  | 0.60 (0.48-0.76) | **0.001** | 0.74 (0.59-0.92) | **0.011** | 0.73 (0.58-0.91) | 0.246 | 0.74 (0.58-0.95) | **0.035** |
| Women | 1.00 (ref) |  | 1.04 (0.82-1.31) |  | 1.11 (0.88-1.39) |  | 0.87 (0.68-1.12) |  | 1.05 (0.80-1.37) |  |
| Incident all cardiovascular disease | | | | | | | | | | |
| Men | 1.00 (ref) |  | 1.01 (0.92-1.12) | 0.494 | 0.98 (0.89-1.08) | 0.667 | 0.96 (0.87-1.06) | 0.599 | 1.00 (0.90-1.12) | 0.548 |
| Women | 1.00 (ref) |  | 0.97 (0.89-1.06) |  | 1.00 (0.92-1.10) |  | 0.93 (0.84-1.02) |  | 1.04 (0.94-1.16) |  |
| Incident aortic stenosis | | | | | | | | | | |
| Men | 1.00 (ref) |  | 1.20 (0.74-1.94) | 0.067 | 1.22 (0.75-1.98) | 0.378 | 1.18 (0.72-1.94) | 0.099 | 1.37 (0.82-2.31) | **0.031** |
| Women | 1.00 (ref) |  | 0.65 (0.42-1.02) |  | 0.92 (0.61-1.39) |  | 0.69 (0.44-1.09) |  | 0.67 (0.40-1.14) |  |
| Incident cardiac failure | | | | | | | | | | |
| Men | 1.00 (ref) |  | 0.88 (0.71-1.09) | 0.111 | 0.85 (0.68-1.07) | 0.495 | 0.95 (0.76-1.18) | 0.628 | 0.94 (0.73-1.20) | 0.413 |
| Women | 1.00 (ref) |  | 1.13 (0.90-1.41) |  | 0.95 (0.75-1.21) |  | 1.02 (0.80-1.29) |  | 1.07 (0.82-1.40) |  |
| Incident myocardial infarction | | | | | | | | | | |
| Men | 1.00 (ref) |  | 0.89 (0.69-1.14) | **0.046** | 0.90 (0.70-1.16) | 0.730 | 0.95 (0.73-1.22) | 0.147 | 0.83 (0.62-1.11) | 0.207 |
| Women | 1.00 (ref) |  | 1.34 (0.98-1.83) |  | 0.97 (0.69-1.36) |  | 1.27 (0.91-1.77) |  | 1.09 (0.75-1.59) |  |
| Incident peripheral arterial disease | | | | | | | | | | |
| Men | 1.00 (ref) |  | 0.88 (0.71-1.11) | 0.431 | 0.96 (0.77-1.20) | 0.846 | 0.89 (0.71-1.13) | 0.617 | 1.04 (0.81-1.34) | 0.669 |
| Women | 1.00 (ref) |  | 1.02 (0.78-1.33) |  | 1.00 (0.75-1.31) |  | 0.98 (0.73-1.30) |  | 1.13 (0.82-1.54) |  |
| Incident stroke | | | | | | | | | | |
| Men | 1.00 (ref) |  | 0.76 (0.60-0.95) | 0.253 | 0.66 (0.53-0.84) | **0.011** | 0.69 (0.55-0.87) | 0.169 | 0.86 (0.67-1.09) | 0.259 |
| Women | 1.00 (ref) |  | 0.90 (0.73-1.11) |  | 0.98 (0.80-1.21) |  | 0.85 (0.69-1.06) |  | 1.02 (0.80-1.29) |  |

P (int) – P value for the interaction term between sex and calcium quintiles.

Adjusted for age, body mass index, systolic blood pressure, low-density lipoprotein, high-density lipoprotein and total cholesterol levels, social class, education level, physical activity, alcohol intake, smoking status, pre-existing comorbidities (hypertension, stroke, myocardial infarction, diabetes), medication and supplement use (aspirin, statins, ACE inhibitors, beta-blockers, angiotensin receptor blockers, vitamin supplement use), dietary intake (total energy intake, fruit and vegetable, vitamin D and calcium intake) and current/former usage of hormone replacement therapy (HRT).

**Supplementary Table 7.** Results of Cox regressions assessing the relationship between quintiles of calcium intake and incident mortality and cardiovascular events in 3261 women having used hormone replacement therapy at the baseline of the EPIC-Norfolk study.

| **Outcome** | **Calcium intake**  **<770 mg/day** | | **Calcium intake**  **771-926 mg/day** | | **Calcium intake**  **927-1073 mg/day** | | **Calcium intake 1074-1254 mg/day** | | **Calcium intake ≥1255 mg/day** | |
| --- | --- | --- | --- | --- | --- | --- | --- | --- | --- | --- |
|  | **HR (95% CI)** | ***P*** | **HR (95% CI)** | ***P*** | **HR (95% CI)** | ***P*** | **HR (95% CI)** | ***P*** | **HR (95% CI)** | ***P*** |
| All-cause mortality | | | | | | | | | | |
| Unadjusted | 1.00 (ref) |  | 0.79 (0.60-1.05) | 0.109 | 0.86 (0.65-1.13) | 0.288 | 0.88 (0.67-1.16) | 0.379 | 0.91 (0.70-1.20) | 0.513 |
| Fully adjusted | 1.00 (ref) |  | **0.70 (0.53-0.94)** | **0.016** | **0.73 (0.55-0.98)** | **0.035** | **0.73 (0.54-0.99)** | **0.043** | 0.72 (0.51-1.02) | 0.069 |
| Cardiovascular mortality | | | | | | | | | | |
| Unadjusted | 1.00 (ref) |  | 0.75 (0.39-1.44) | 0.388 | 1.18 (0.66-2.10) | 0.582 | 1.17 (0.66-2.10) | 0.587 | 0.94 (0.51-1.73) | 0.836 |
| Fully adjusted | 1.00 (ref) |  | 0.64 (0.33-1.26) | 0.199 | 1.01 (0.54-1.89) | 0.971 | 0.94 (0.48-1.84) | 0.863 | 0.81 (0.37-1.79) | 0.610 |
| Incident all cardiovascular disease | | | | | | | | | | |
| Unadjusted | 1.00 (ref) |  | 0.95 (0.81-1.12) | 0.565 | 1.03 (0.88-1.20) | 0.742 | 0.91 (0.77-1.07) | 0.242 | 0.99 (0.85-1.16) | 0.917 |
| Fully adjusted | 1.00 (ref) |  | 0.83 (0.70-0.98) | 0.027 | 0.93 (0.79-1.10) | 0.391 | 0.84 (0.70-1.01) | 0.061 | 0.93 (0.76-1.13) | 0.468 |
| Incident aortic stenosis | | | | | | | | | | |
| Unadjusted | 1.00 (ref) |  | 0.49 (0.20-1.21) | 0.123 | 0.63 (0.27-1.47) | 0.287 | 0.70 (0.31-1.58) | 0.392 | **0.28 (0.09-0.85)** | **0.024** |
| Fully adjusted | 1.00 (ref) |  | 0.45 (0.18-1.15) | 0.095 | 0.60 (0.24-1.49) | 0.270 | 0.68 (0.26-1.77) | 0.431 | **0.25 (0.06-0.95)** | **0.043** |
| Incident cardiac failure | | | | | | | | | | |
| Unadjusted | 1.00 (ref) |  | 1.14 (0.67-1.95) | 0.631 | 1.31 (0.78-2.21) | 0.305 | 1.21 (0.72-2.06) | 0.469 | 1.03 (0.59-1.78) | 0.923 |
| Fully adjusted | 1.00 (ref) |  | 1.11 (0.64-1.92) | 0.719 | 1.29 (0.74-2.24) | 0.374 | 1.17 (0.65-2.11) | 0.608 | 1.13 (0.57-2.25) | 0.730 |
| Incident myocardial infarction | | | | | | | | | | |
| Unadjusted | 1.00 (ref) |  | 0.78 (0.40-1.53) | 0.470 | 0.68 (0.33-1.37) | 0.278 | 1.04 (0.56-1.95) | 0.900 | **0.41 (0.18-0.95)** | **0.037** |
| Fully adjusted | 1.00 (ref) |  | 0.69 (0.34-1.40) | 0.309 | 0.65 (0.31-1.38) | 0.266 | 0.99 (0.48-2.07) | 0.988 | 0.40 (0.14-1.13) | 0.085 |
| Incident peripheral arterial disease | | | | | | | | | | |
| Unadjusted | 1.00 (ref) |  | 0.81 (0.49-1.34) | 0.415 | 0.97 (0.60-1.56) | 0.893 | **0.55 (0.31-0.96)** | **0.035** | 0.81 (0.49-1.34) | 0.418 |
| Fully adjusted | 1.00 (ref) |  | 0.83 (0.49-1.38) | 0.471 | 1.10 (0.66-1.83) | 0.727 | 0.68 (0.37-1.26) | 0.221 | 1.09 (0.57-2.08) | 0.793 |
| Incident stroke | | | | | | | | | | |
| Unadjusted | 1.00 (ref) |  | 0.78 (0.49-1.25) | 0.304 | 0.88 (0.56-1.38) | 0.575 | 1.03 (0.67-1.59) | 0.891 | 0.98 (0.63-1.53) | 0.942 |
| Fully adjusted | 1.00 (ref) |  | 0.71 (0.44-1.14) | 0.152 | 0.77 (0.48-1.24) | 0.284 | 0.84 (0.51-1.37) | 0.485 | 0.79 (0.45-1.39) | 0.412 |

Adjusted for age, body mass index, systolic blood pressure, low-density lipoprotein, high-density lipoprotein and total cholesterol levels, social class, education level, physical activity, alcohol intake, smoking status, pre-existing comorbidities (hypertension, stroke, myocardial infarction, diabetes), medication and supplement use (aspirin, statins, ACE inhibitors, beta-blockers, angiotensin receptor blockers, vitamin supplement use) and dietary intake (total energy intake, fruit and vegetable, vitamin D and calcium intake).

**Supplementary Table 8.** Results of Cox regressions assessing the relationship between quintiles of calcium intake and incident mortality and cardiovascular events in 15,396 men and women without prevalent hypertension or diabetes mellitus at the baseline of the EPIC-Norfolk study.

| **Outcome** | **Calcium intake**  **<770 mg/day** | | **Calcium intake**  **771-926 mg/day** | | **Calcium intake**  **927-1073 mg/day** | | **Calcium intake 1074-1254 mg/day** | | **Calcium intake ≥1255 mg/day** | |
| --- | --- | --- | --- | --- | --- | --- | --- | --- | --- | --- |
|  | **HR (95% CI)** | ***P*** | **HR (95% CI)** | ***P*** | **HR (95% CI)** | ***P*** | **HR (95% CI)** | ***P*** | **HR (95% CI)** | ***P*** |
| All-cause mortality | | | | | | | | | | |
| Unadjusted | 1.00 (ref) |  | 1.00 (0.90-1.10) | 0.967 | 1.09 (0.98-1.20) | 0.104 | 1.02 (0.93-1.13) | 0.655 | 1.00 (0.90-1.10) | 0.939 |
| Fully adjusted | 1.00 (ref) |  | 0.93 (0.84-1.03) | 0.162 | 1.00 (0.90-1.11) | 0.995 | **0.89 (0.80-1.00)** | **0.050** | 0.97 (0.85-1.10) | 0.616 |
| Cardiovascular mortality | | | | | | | | | | |
| Unadjusted | 1.00 (ref) |  | 0.94 (0.78-1.14) | 0.542 | 1.04 (0.86-1.25) | 0.716 | 1.03 (0.85-1.24) | 0.786 | 0.92 (0.76-1.12) | 0.416 |
| Fully adjusted | 1.00 (ref) |  | 0.88 (0.72-1.06) | 0.183 | 0.93 (0.76-1.14) | 0.482 | 0.87 (0.70-1.07) | 0.180 | 0.90 (0.70-1.14) | 0.378 |
| Incident all cardiovascular disease | | | | | | | | | | |
| Unadjusted | 1.00 (ref) |  | 1.02 (0.95-1.10) | 0.602 | 1.07 (0.99-1.15) | 0.076 | 1.05 (0.98-1.13) | 0.182 | 1.02 (0.95-1.10) | 0.594 |
| Fully adjusted | 1.00 (ref) |  | 0.97 (0.90-1.05) | 0.451 | 1.01 (0.94-1.09) | 0.747 | 0.98 (0.90-1.06) | 0.591 | 1.01 (0.92-1.11) | 0.886 |
| Incident aortic stenosis | | | | | | | | | | |
| Unadjusted | 1.00 (ref) |  | 0.78 (0.54-1.12) | 0.176 | 0.96 (0.68-1.35) | 0.807 | 0.93 (0.65-1.31) | 0.672 | 0.90 (0.63-1.27) | 0.537 |
| Fully adjusted | 1.00 (ref) |  | 0.75 (0.51-1.09) | 0.126 | 0.90 (0.62-1.30) | 0.566 | 0.85 (0.57-1.26) | 0.423 | 0.88 (0.56-1.39) | 0.585 |
| Incident cardiac failure | | | | | | | | | | |
| Unadjusted | 1.00 (ref) |  | 0.96 (0.81-1.15) | 0.691 | 0.91 (0.76-1.10) | 0.330 | 1.03 (0.86-1.23) | 0.745 | 0.94 (0.78-1.12) | 0.488 |
| Fully adjusted | 1.00 (ref) |  | 0.93 (0.77-1.11) | 0.410 | 0.90 (0.74-1.09) | 0.280 | 0.98 (0.80-1.19) | 0.832 | 1.04 (0.82-1.31) | 0.748 |
| Incident myocardial infarction | | | | | | | | | | |
| Unadjusted | 1.00 (ref) |  | 1.07 (0.85-1.34) | 0.565 | 1.07 (0.85-1.34) | 0.560 | 1.19 (0.95-1.48) | 0.129 | 0.95 (0.75-1.20) | 0.643 |
| Fully adjusted | 1.00 (ref) |  | 1.03 (0.82-1.30) | 0.780 | 1.03 (0.81-1.31) | 0.803 | 1.12 (0.87-1.44) | 0.380 | 0.94 (0.70-1.27) | 0.698 |
| Incident peripheral arterial disease | | | | | | | | | | |
| Unadjusted | 1.00 (ref) |  | 1.04 (0.86-1.27) | 0.686 | 1.08 (0.89-1.31) | 0.455 | 0.98 (0.81-1.20) | 0.880 | 1.05 (0.86-1.27) | 0.633 |
| Fully adjusted | 1.00 (ref) |  | 1.01 (0.82-1.23) | 0.943 | 1.07 (0.87-1.31) | 0.549 | 0.97 (0.78-1.21) | 0.790 | 1.11 (0.86-1.42) | 0.420 |
| Incident stroke | | | | | | | | | | |
| Unadjusted | 1.00 (ref) |  | 0.84 (0.71-1.00) | 0.057 | 0.91 (0.76-1.07) | 0.257 | 0.88 (0.74-1.04) | 0.139 | 0.98 (0.82-1.15) | 0.771 |
| Fully adjusted | 1.00 (ref) |  | **0.78 (0.66-0.94)** | **0.007** | **0.81 (0.67-0.97)** | **0.024** | **0.75 (0.62-0.91)** | **0.003** | 0.90 (0.72-1.12) | 0.330 |

Adjusted for age, sex, body mass index, systolic blood pressure, low-density lipoprotein, high-density lipoprotein and total cholesterol levels, social class, education level, physical activity, alcohol intake, smoking status, pre-existing comorbidities (hypertension, stroke, myocardial infarction, diabetes), medication and supplement use (aspirin, statins, ACE inhibitors, beta-blockers, angiotensin receptor blockers, vitamin supplement use), dietary intake (total energy intake, fruit and vegetable, vitamin D and calcium intake) and current/former usage of hormone replacement therapy (HRT).

25,639 participants in EPIC-Norfolk cohort

5,211 participants excluded due to missing data:*

- Calcium supplementation (n=2,619)
- Vitamin supplementation (n=101)
- Food frequency questionnaire (n=890)
- Total cholesterol levels (n=1,768)
- LDL/HDL cholesterol levels (n=2,569)
- Social class (n=570)
- Physical activity levels (n=1)
- Systolic blood pressure (n=60)
- Body mass index (n=57)

19,838 participants

1,870 participants excluded due to prevalent cancer or cardiovascular disease at baseline:

- Cancer (n=1,091)
- Stroke (n=257)
- Myocardial infarction (n=611)

17,968 participants included in the analysis

**Supplementary Figure 1.** Flow diagram of participants included in the analysis

*Missing data were defined as no value entered into the database. Some participants had multiple missing data, hence values shown sum to more than the number of participants excluded.

**Supplementary Figure 2**. Results of the multivariable Cox regressions assessing the relationship between daily calcium intake as a continuous variable and incident mortality and cardiovascular events in 17,968 men and women of the EPIC-Norfolk study.

Log Hazard ratios and respective 95% confidence intervals are represented by the blue line with grey shadowing. The dotted red line represents the reference line (log HR = 0). The overlaying blue bar chart displays the distribution of daily calcium intake in the included cohort.

Adjusted for age, sex, body mass index, systolic blood pressure, low-density lipoprotein, high-density lipoprotein and total cholesterol levels, social class, education level, physical activity, alcohol intake, smoking status, pre-existing comorbidities (hypertension, stroke, myocardial infarction, diabetes), medication and supplement use (aspirin, statins, ACE inhibitors, beta-blockers, angiotensin receptor blockers, vitamin supplement use), dietary intake (total energy intake, fruit and vegetable, vitamin D and calcium intake) and current/former usage of hormone replacement therapy (HRT).

CVD – all cardiovascular disease; PAD – peripheral arterial disease

**Supplementary Figure 3.** Meta-analysis (forest plot) of the association between dietary calcium intake (highest compared to the lowest level, mg/day) and risk of all-cause mortality stratified by sex.

**Supplementary Figure 4.**  Meta-analysis (forest plot) of the association between dietary calcium intake (highest compared to the lowest level, mg/day) and risk of CVD mortality stratified by sex.
